# Supplementary material for: On-surface synthesis of enetriynes
Source: Nat Commun. 2023 Mar 6;14:1255. doi: 10.1038/s41467-023-36828-y (PMC9988975; doi:10.1038/s41467-023-36828-y)
Supplement: Supplementary file 1 — Supplementary Information [file 41467_2023_36828_MOESM1_ESM.pdf]

Supplementary Information

On-surface synthesis of enetriynes

Nan Cao[1,2,+], Biao Yang[1,+,*], Alexander Riss[1], Johanna Rosen[2], Jonas Björk[2,*], Johannes V. Barth[1]

[1] *Physics Department E20, Technical University of Munich, D-85748 Garching, (Germany)*

[2] *Department of Physics, Chemistry and Biology, IFM, Linköping University, 58183 Linköping (Sweden)*

[+]These authors contributed equally.

*Email: biao.yang@tum.de, jonas.bjork@liu.se

Table of content

Supplementary Figure 1. XPS spectra of C 1s and O 1s core level for EHBP deposited on the Ag(100) surface held at 150 K and upon warming up to RT.

Supplementary Figure 2. Large-scale STM images of sample surfaces at different preparation stages.

Supplementary Figure 3. EHBP self-assembly and corresponding structural model from computational modeling for layer formed at RT.

Supplementary Figure 4. STM image simulation of an organometallic dimer.

Supplementary Figure 5. Comparison of the experimental and simulated bias-dependent STM data of the organometallic dimer array.

Supplementary Figure 6. STM and AFM images of a dimer pair showing different interactions in the vicinity of terminal oxygen.

Supplementary Figure 7. Detailed STM and AFM imaging and analysis of two enetriyne tetramers.

Supplementary Figure 8. Extended assembly structure of the tetramer products.

Supplementary Figure 9. Detailed STM and AFM imaging and analysis of side products.

Supplementary Figure 10. Statistical analysis of enyne reaction products.

Supplementary Figure 11. C 1s core level photoemission spectra of molecular layers at different reaction stages.

Supplementary Figure 12. Initial signs of the formation of enetriynes.

Supplementary Figure 13. Modeled alternative reaction pathways of the addition reaction to form final tetrameric enetriyne products.

Supplementary Figure 14. Reaction pathways and energy profiles for the cleavage of an organometallic dimer.

Supplementary Figure 15. DFT modelling for an alternative reaction pathway of the first addition reaction.

Supplementary Figure 16. Temperature-dependent calculation of the rate-limiting step in the reaction pathway.

Supplementary Figure 17. Reaction scenario of EBP on Ag(100).

Supplementary Figure 18. Reaction scenario of DEBP on Ag(100).

Supplementary Figure 19. Comparative experiment of annealing an EHBP layer on Ag(100) without previous O_2 exposure.

Supplementary Figure 20. Reaction scenario of EHBP on Ag(111).

Supplementary references

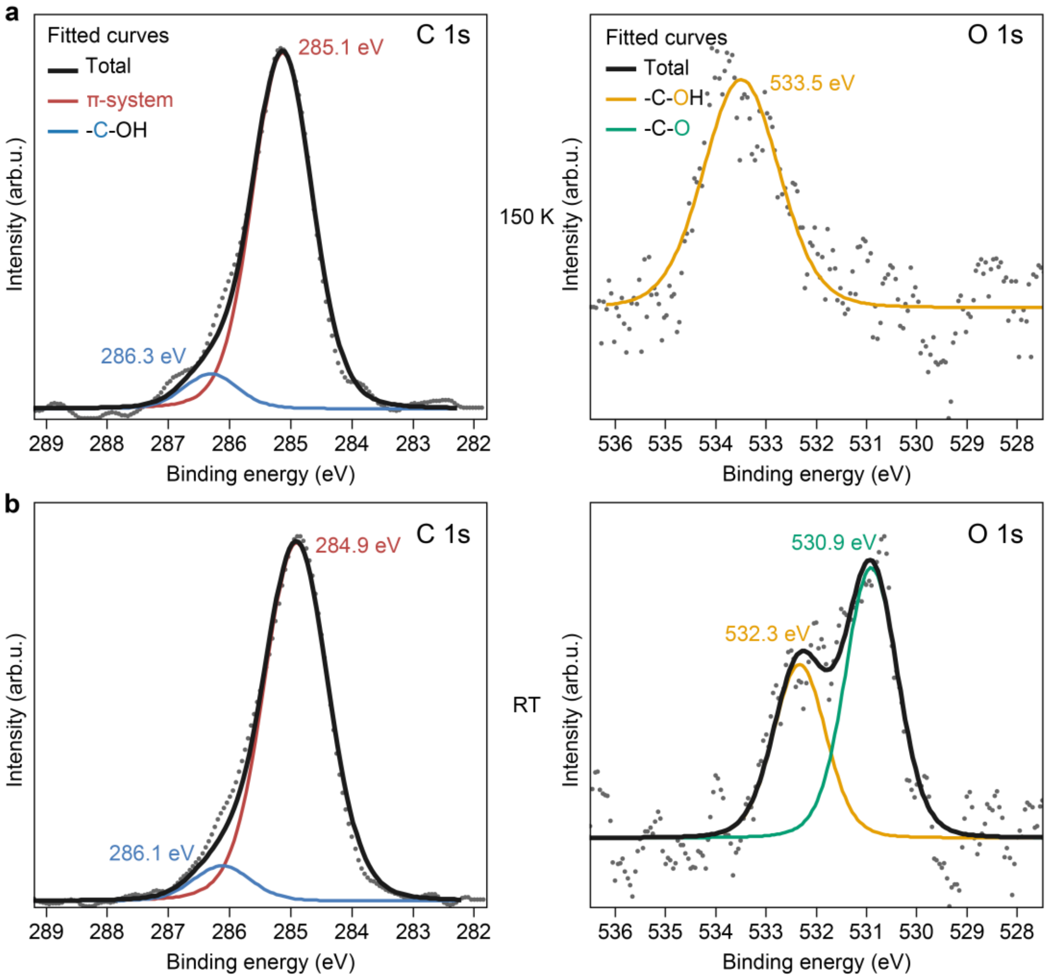

Supplementary Figure 1. XPS spectra of C 1s and O 1s core level for EHBP deposited on the Ag(100) surface held at 150 K and upon warming up to RT. (a) After deposition of EHBP on Ag(100) surfaces with a medium coverage at 150 K, the O 1s region shows a single peak at 533.5 eV, which is related to the oxygen of -OH (right panel)[1-4]. (b) Upon warming up to RT, the O 1s spectra are splitting into two peaks at BE of 532.3 eV and 530.9 eV (right panel), which are assigned to the oxygen of -OH and -C-O, respectively[1-4]. Note that the shifts of the O 1s signal of -OH could be related to the changes in the intermolecular interactions at different temperatures. Correspondingly, the C 1s spectra also display a slight downward shift. The C 1s spectra of the sample at 150 K present one main peak (left panel in Supplementary Figure 1a). It can be fitted with two peaks, the high BE of 286.3 eV is the typical sign of -C-OH, and the low BE of 285.1 eV is assigned to the π-system consisting of phenyl and alkynyl groups. The peak intensity has a ratio of ~1:11.5, close to the stoichiometric value of 1:13. After warming the sample to RT (left panel in Supplementary Figure 1b), the two C 1s peaks appear at lower BE of 286.1 eV and 284.9 eV, respectively. Therefore we propose that EHBP molecules remain pristine on the Ag(100) surface at 150 K; partial dehydrogenation occurs upon annealing to RT, as the suggested chemical model shown in Figure 2 in the main text. For the spectra fitting, a Shirley (C 1s) or linear (O 1s) baseline were employed for background subtraction from the raw data (gray dots in the plots). Source data are provided as a Source Data file.

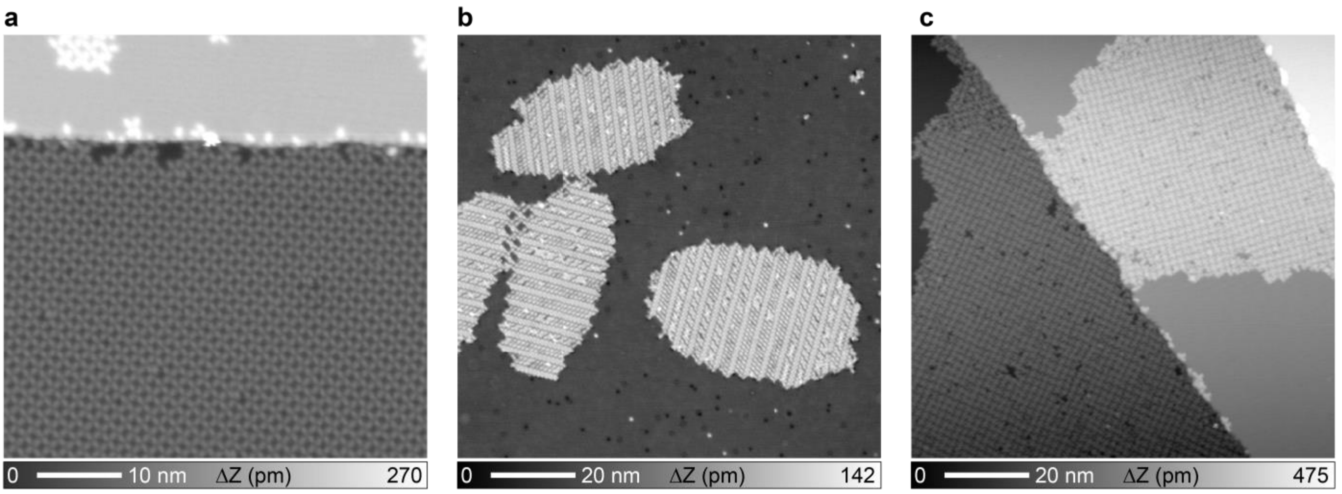

Supplementary Figure 2. Large-scale STM images of sample surfaces at different preparation stages. (a) Following deposition on the pristine Ag(100) substrate held at RT. (b) After O_2 exposure (~450 L) at 300 K. (c) Upon annealing the oxygen-treated sample at 520 K. Measurement parameters: (a) and (b) I_t = 100 pA, V_s = -100 mV, (c) I_t = 100 pA, V_s = -500 mV.

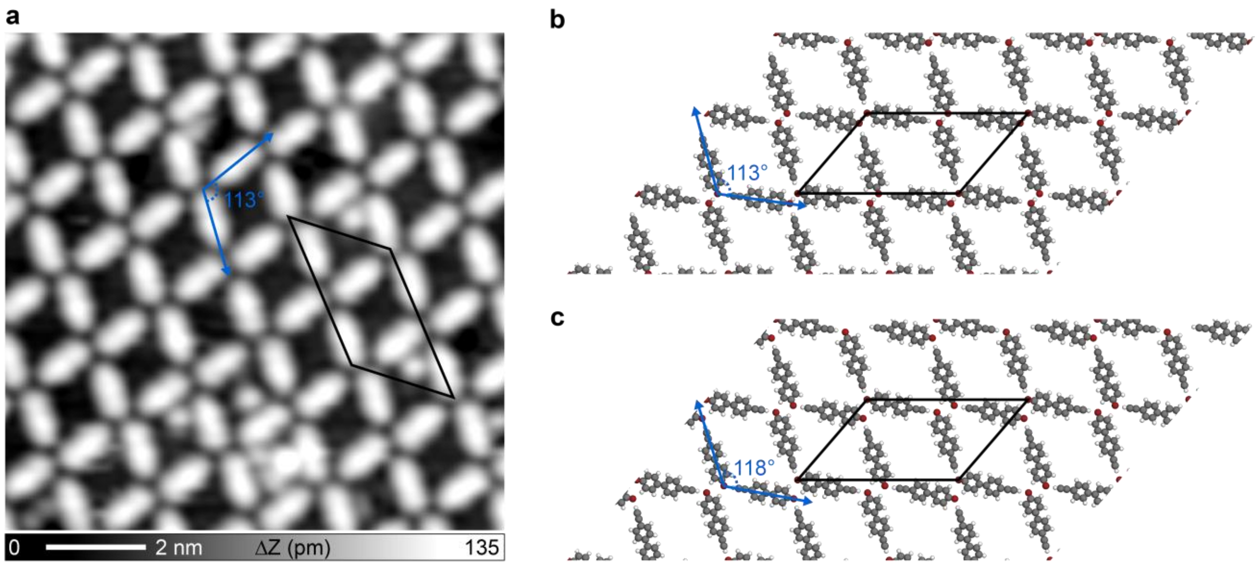

Supplementary Figure 3. EHBP self-assembly and corresponding structural model from computational modeling for layer formed at RT. (a) High-resolution STM image of network formed at RT (Measurement parameters: I_t = 10 pA, V_s = 100 mV). Optimized structural models from DFT calculations with half-dehydrogenated hydroxyl groups and fully dehydrogenated hydroxyl groups are shown in (b) and (c), respectively. Black framework shows the unit cell of the self-assembly structure, the unit cell of structural models in (b) and (c) are identical to the experimental value in (a). Blue arrows display the direction of the molecular axis. The angle spanned by the molecular axes in (b) is comparable with the angle determined from the orientation in STM data (a). This agrees well with the chemical examination of the hydroxyl moiety by XPS (Supplementary Figure 1). Source data are provided as a Source Data file.

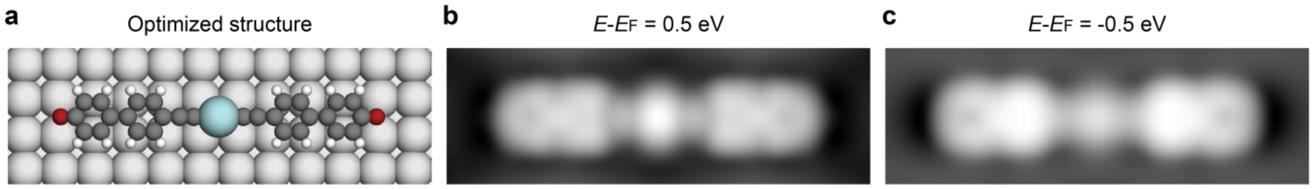

Supplementary Figure 4. STM image simulation of an organometallic dimer. (a) DFT optimized structural model of an organometallic dimer on the most stable adsorption position. Simulated STM image of a single organometallic dimer at 0.5 eV in (b) and -0.5 eV in (c). Source data are provided as a Source Data file.

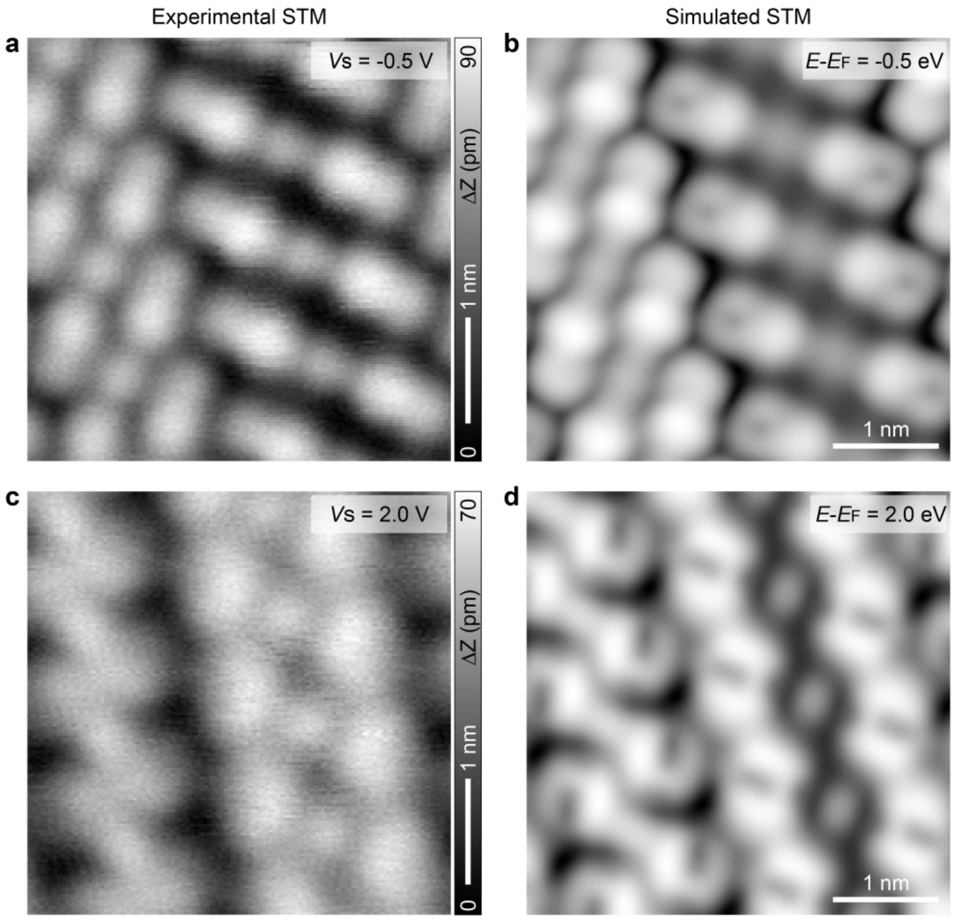

Supplementary Figure 5. Comparison of the experimental and simulated bias-dependent STM data of the organometallic dimer array. (a) and (c) Experimental STM images of the organometallic dimer array on Ag(100). (b) and (d) Simulated STM images corresponding to (a) and (c), respectively. Measurement parameters: (a) I_t = 10 pA, V_s = -500 mV; (c) I_t = 10 pA, V_s = 2 V.

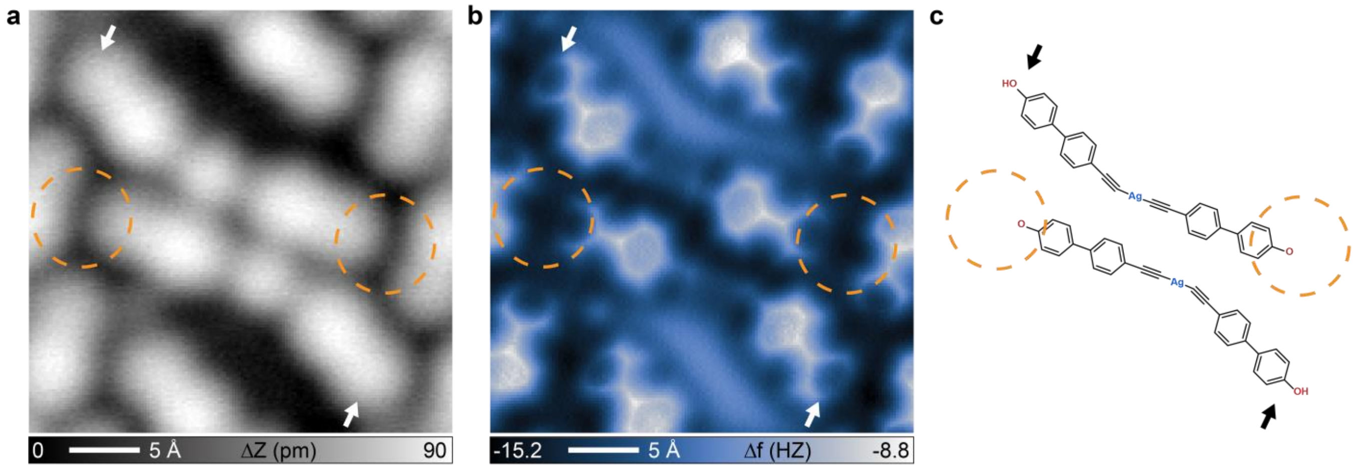

Supplementary Figure 6. STM and AFM images of a dimer pair showing different interactions in the vicinity of terminal oxygen. (a) STM topology of a dimer pair and (b) AFM inspection of the same area. (c) Corresponding chemical structure of the dimers in (a) and (b). The two ends marked by orange circles are dehydrogenated hydroxyl entities, which point towards the biphenyl backbone of the adjacent dimer, analogous to the situation in Figure 3d in the main text. The two other ends bend outward (shown by white arrows in (a) and (b), black arrows in (c)) and are assigned to hydroxyl entities with relatively reduced contrast, as compared to dehydrogenated ones. Measurement parameters: (a) I_t = 10 pA, V_s = 100 mV; (b) V_s = 0 V, constant height mode.

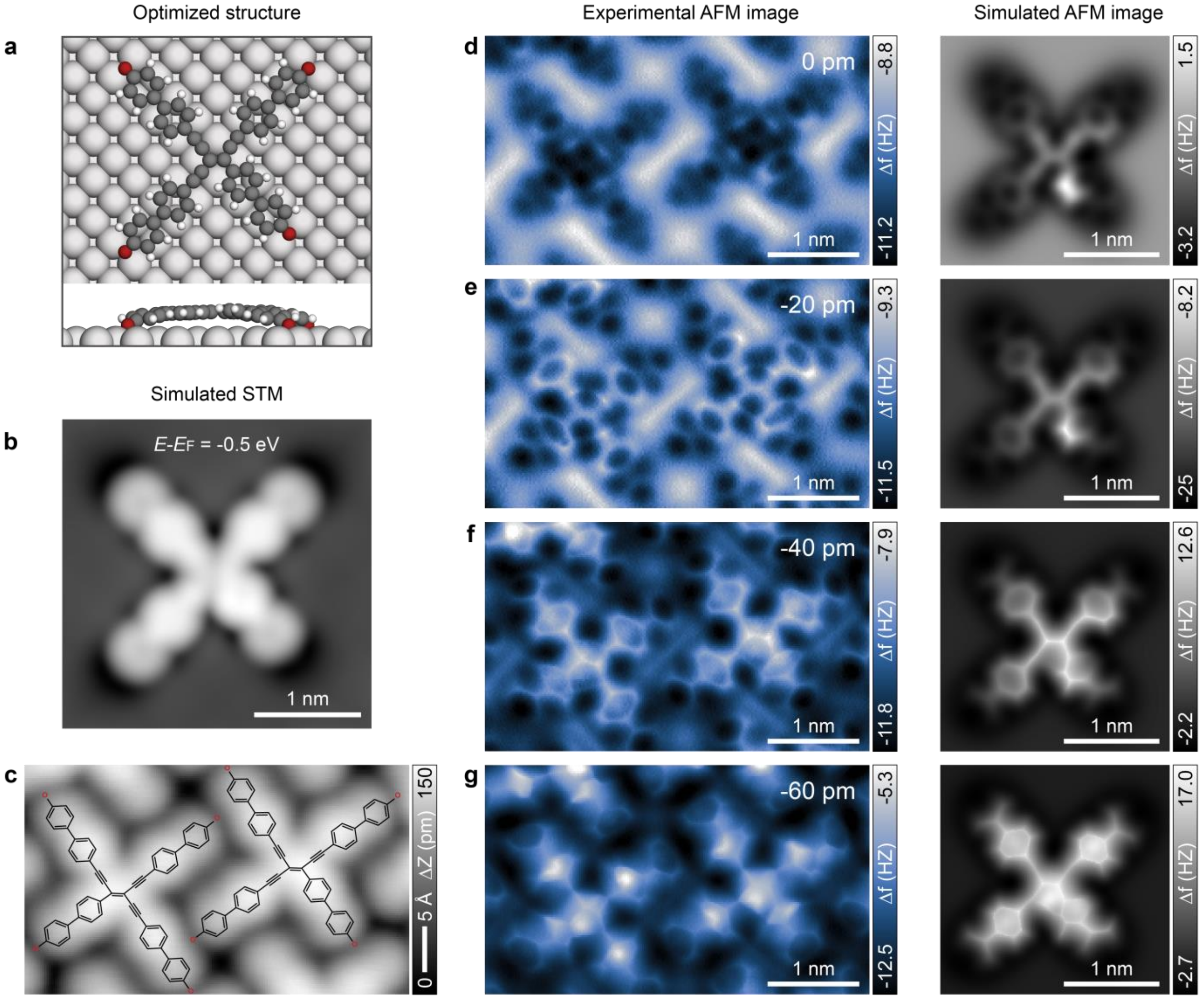

Supplementary Figure 7. Detailed STM and AFM imaging and analysis of two enetriyne tetramers. (a) DFT optimized structural model of a tetramer for image simulations. (b) STM simulation of the single tetramer. (c) STM image of two tetramers superimposed with the corresponding chemical structure (Measurement parameters: I_t = 10 pA, V_s = 100 mV). (d)-(g) Experimental and simulated AFM images at a series of decreasing tip-sample distances, as indicated by labelled negative tip heights (Measurement parameters: V_s = 0 V, constant height mode). Notably the AFM data at different tip heights shows additional features of enetriynes. The bright line-shape feature of alkynyl bonds are obvious experimentally at higher tip height (Supplementary Figure 7d and 7e), while the simulation does not reproduce them when electrostatic effects are not taken into account. One can see the distortion of phenyl rings at lower tip heights (Supplementary Figure 7f and 7g), in accordance with simulations. In addition, terminal oxygen of each enetriyne face the middle of the biphenyl backbone of adjacent molecules. Source data are provided as a Source Data file.

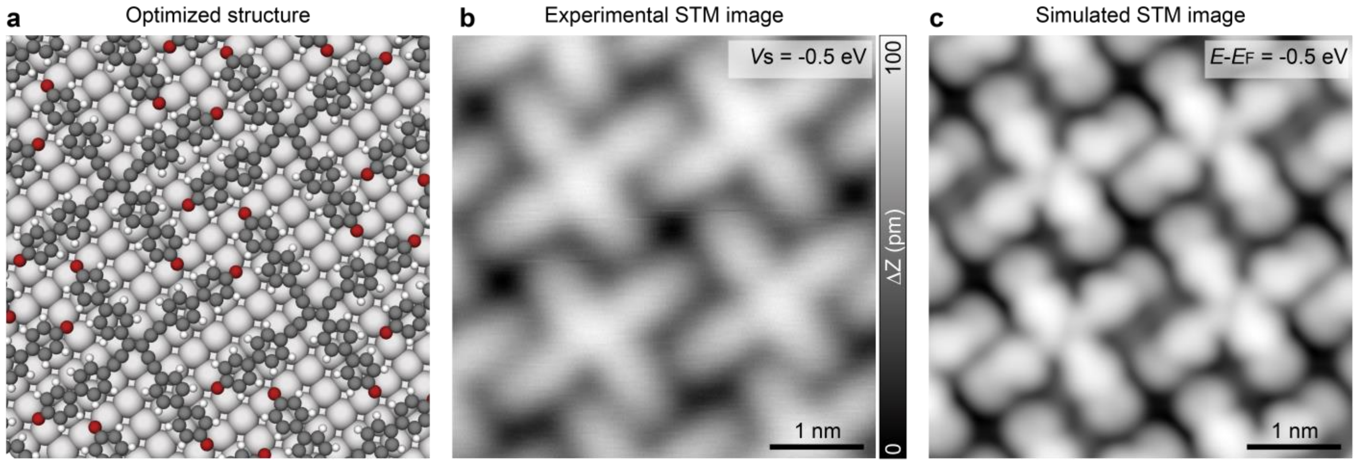

Supplementary Figure 8. Extended assembly structure of enetriyne tetramer products. (a) DFT optimized structural model shows the interactions between enetriyne tetramers. (b) Experimental STM image of four adjacent tetramers (Measurement parameters: I_t = 10 pA, V_s = -500 mV). (c) The corresponding simulated STM image. Source data are provided as a Source Data file.

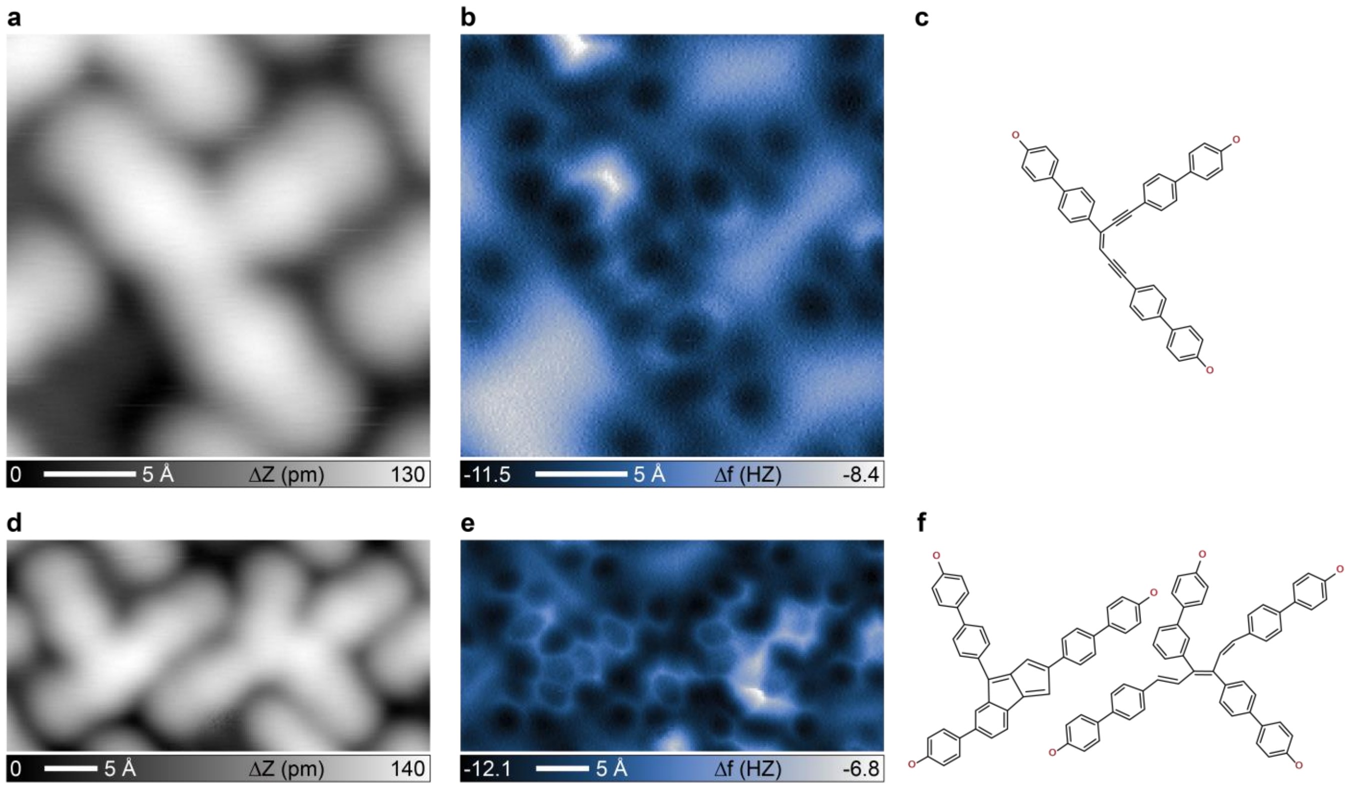

Supplementary Figure 9. Detailed STM and AFM imaging and analysis of side products. (a)-(b) STM and AFM images of an enediyne (corresponding to the area marked by the red square in Supplementary Figure 10c) and chemical structure in (c). (d)-(e) STM and AFM images of a trimeric species and a deformed tetrameric species (corresponding to the area marked by an orange rectangle in Supplementary Figure 10c) and their hypothesized chemical structures in (f). Measurement parameters: (a) and (d) I_t = 10 pA, V_s = 100 mV; (b) and (e): V_s = 0 V, constant height mode.

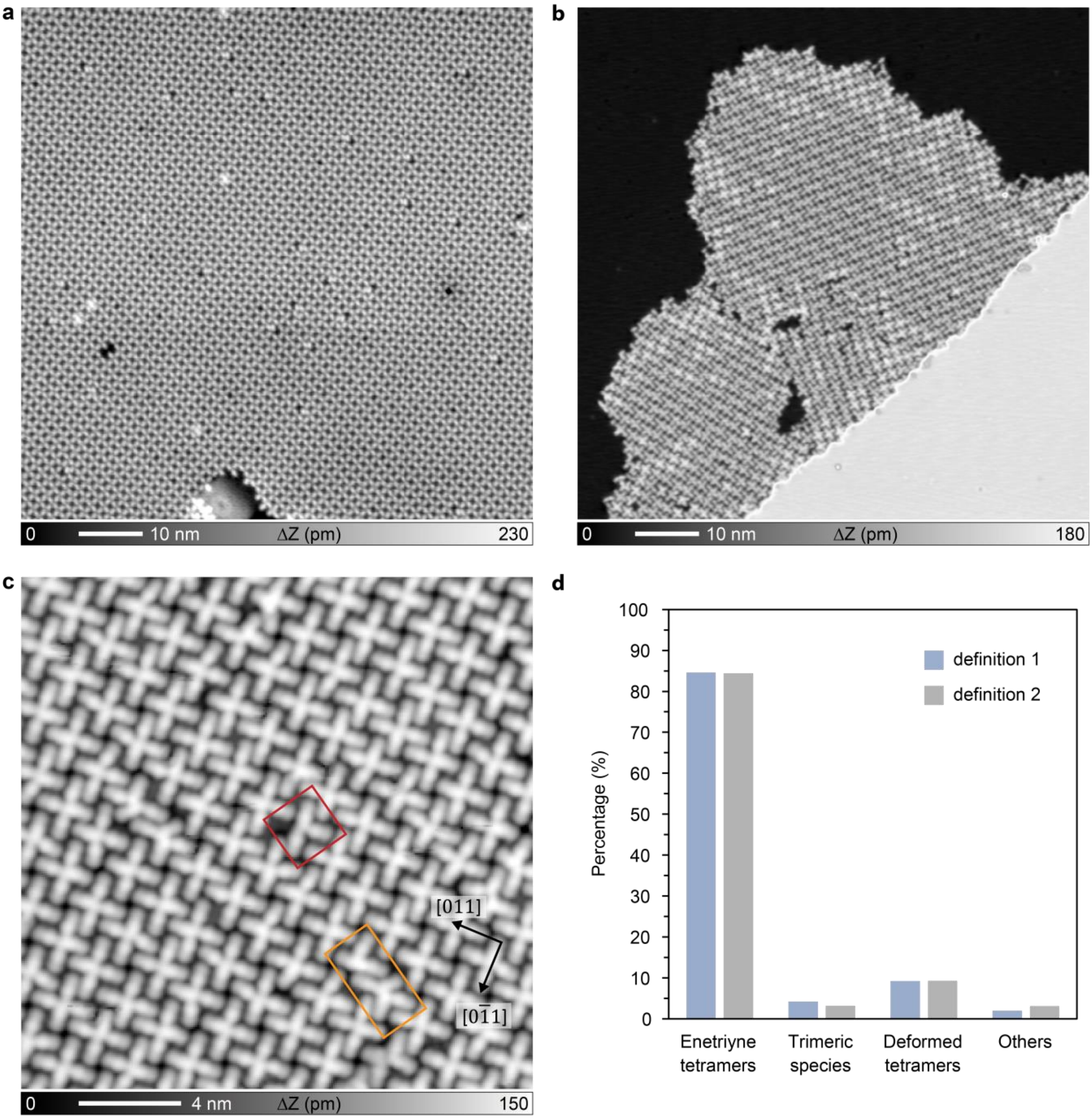

Supplementary Figure 10. Statistical analysis of enyne reaction products. (a) and (b) Large-scale STM images of the structures upon initial RT-deposition and after annealing substrate at 520 K. (c) High-resolution STM image of the products. (d) Statistical distribution of different products on several high-resolution large-scale STM images. Counting more than 2000 products, the yield (*definition 1*) of enetriynes, simply defined as the proportion of enetriynes to all products obtained on the sample, is determined to ~85%. Side products are mainly trimeric species, deformed tetramers, and unidentified derivatives, with ratios of 4%, 9% and 2%, respectively. Another possible definition (*definition 2*) of the yield would be the proportion of monomers for forming the enetriynes to all monomer reactants. We have also estimated the yield in this way and came up with ~84%. Measurement parameters: (a) I_t = 100 pA, V_s = -100 mV; (b) I_t = 100 pA, V_s = -500 mV and (c) I_t = 10 pA, V_s = 100 mV. Source data are provided as a Source Data file.

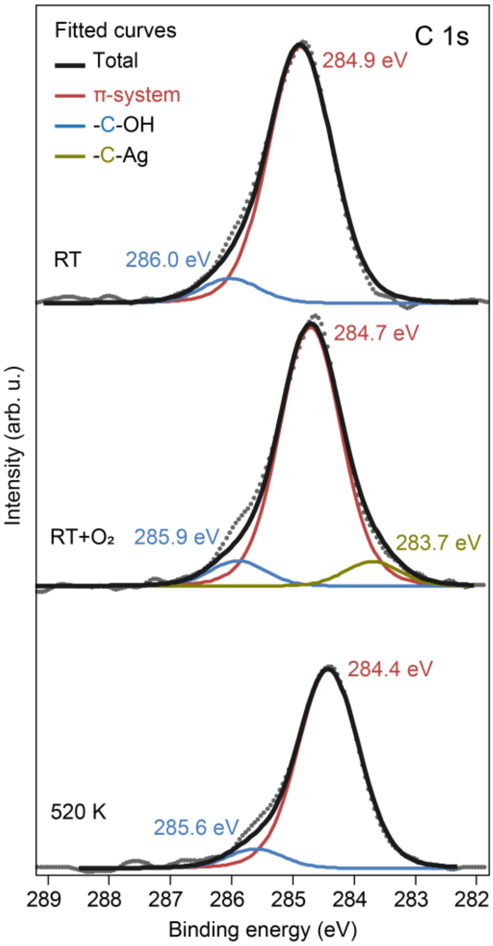

Supplementary Figure 11. C 1s core level photoemission spectra of molecular layers at different reaction stages, complementing the O 1s analysis (see Figure 5 in main text). At RT, the C 1s region can be fitted into two peaks at BEs of 286.0 eV and 284.9 eV, respectively – very similar to the one depicted in Supplementary Figure 1. After O_2 exposure, a new component appears at the low BE region of 283.7 eV, attributed to C-Ag formation, in agreement with previous studies[5]. The slight shift of the main peak is tentatively associated with changes in the intermolecular interactions due to dehydrogenation of hydroxyl groups. This result agrees well with the experimentally observed alkynyl-Ag-alkynyl organometallic dimers. The peak area has a ratio of ~1:11:1 from high to low BE, nearly matching the stoichiometry value 1:12:1. Upon annealing at 520 K, the C-Ag contribution disappears, reflecting the dissociation of Ag adatoms from products. Hereby the shift of the main peak of C 1s from 284.7 eV to 284.4 eV is ascribed to the high enetriyne conjugation. Gray dots in the plots denote the Shirley-background-subtracted XPS data. Source data are provided as a Source Data file.

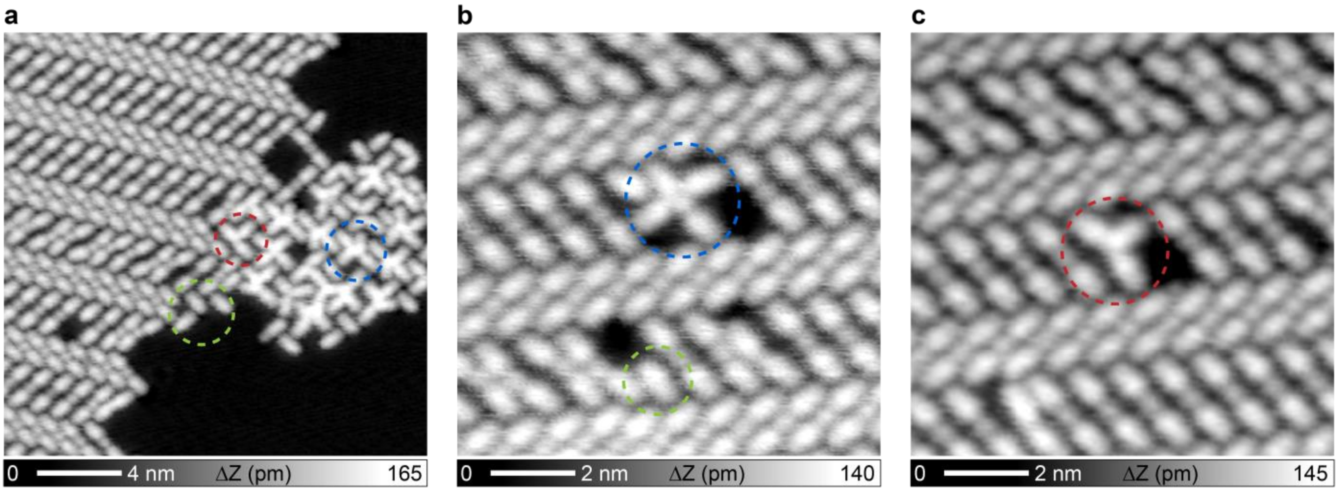

Supplementary Figure 12. Onset of the enetriyne formation upon mild annealing at 450 K. (a) STM image domain edge of organometallic dimers network. Broken dimers, trimeric and tetrameric species exist (marked by green, red and blue dashed circles, respectively). (b) Close-up view of tetrameric species in an organometallic dimer region, implying a possible intermediate state with Ag adatom not removed from the tetramer. (c) Trimeric species embedded in an organometallic dimers region, presumably representing an enediyne derivative. Measurement parameters: (a) I_t = 100 pA, V_s = -100 mV, (b) and (c) I_t = 100 pA, V_s = -500 mV.

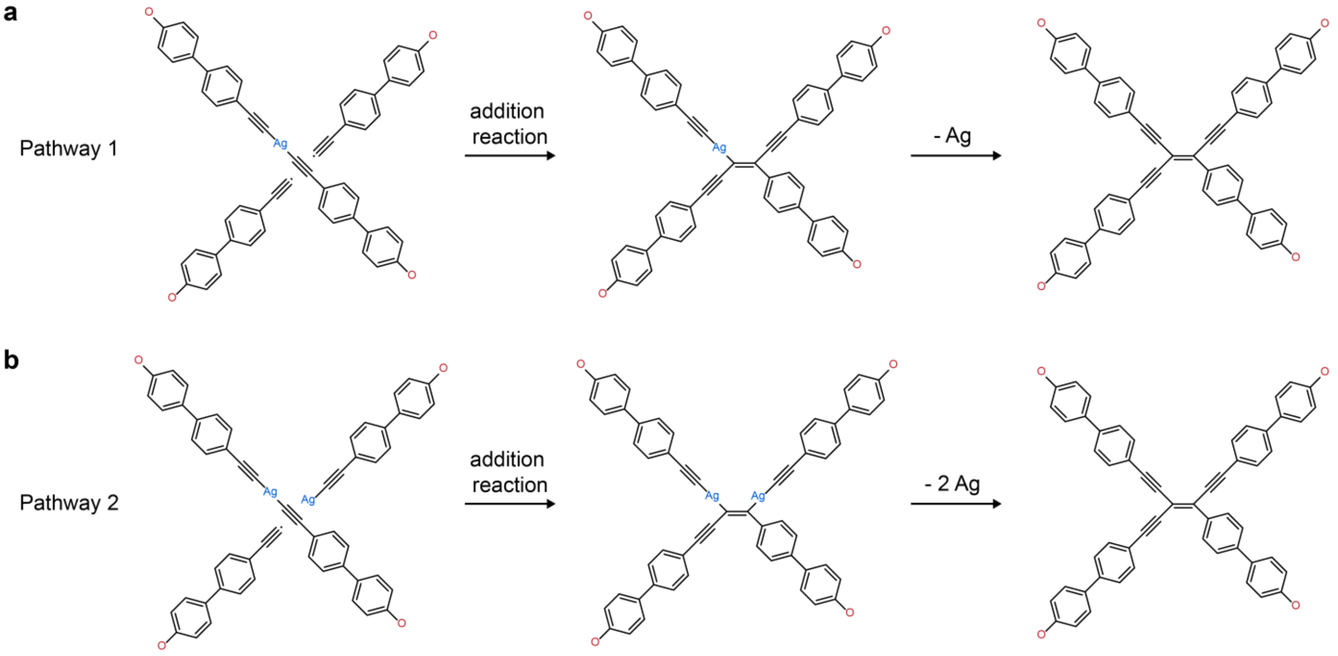

Supplementary Figure 13. Modeled alternative reaction pathways of the addition reaction to form final tetrameric enetriyne products. (a) Reaction pathway 1, the reaction proceeds firstly with addition reactions and then one Ag adatom removal, corresponding to the detailed pathway in Figure 6 in the main text. (b) An alternative reaction pathway 2 starts with addition reactions, whereas further two Ag adatoms removal.

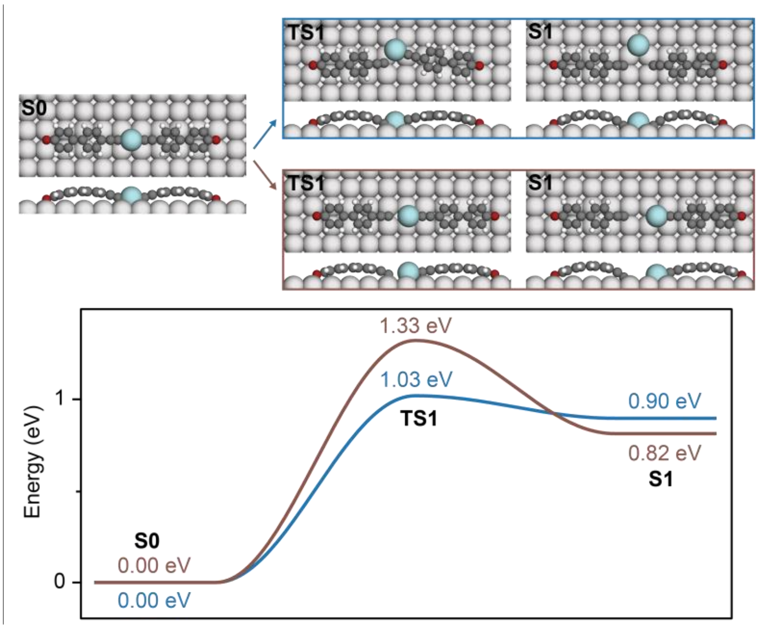

Supplementary Figure 14. Reaction pathways and energy profiles for the cleavage of an organometallic dimer. The reaction pathway traced in blue is the most favorable, whereby the incorporated Ag atom is fully removed from alkynyl groups, with a barrier of 1.03 eV. For the alternative reaction pathway (brown line) the Ag atom remains attached to one of the EHBP entities upon dimer cleavage, which process is associated with an energy barrier of 1.33 eV. Source data are provided as a Source Data file.

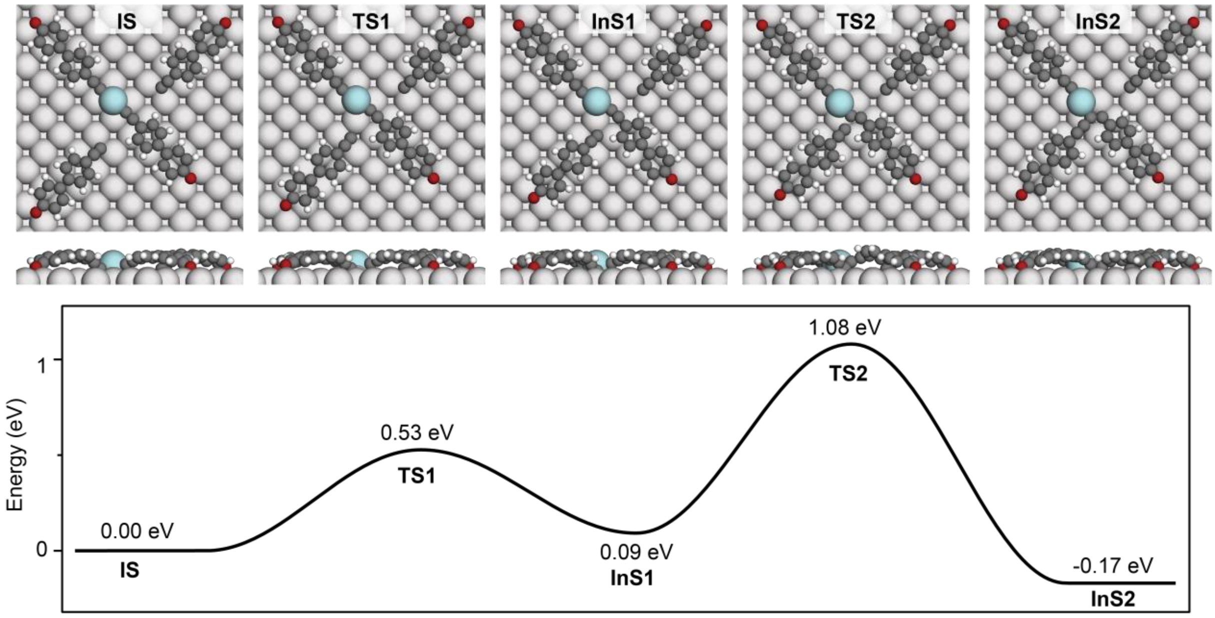

Supplementary Figure 15. Alternative reaction pathway of the first addition reaction with an energy barrier of 0.99 eV. The reaction pathway described in the main text exhibits a similar reaction barrier of 0.98 eV. At the experimental reaction temperature of 520 K, both reaction pathways can occur. Source data are provided as a Source Data file.

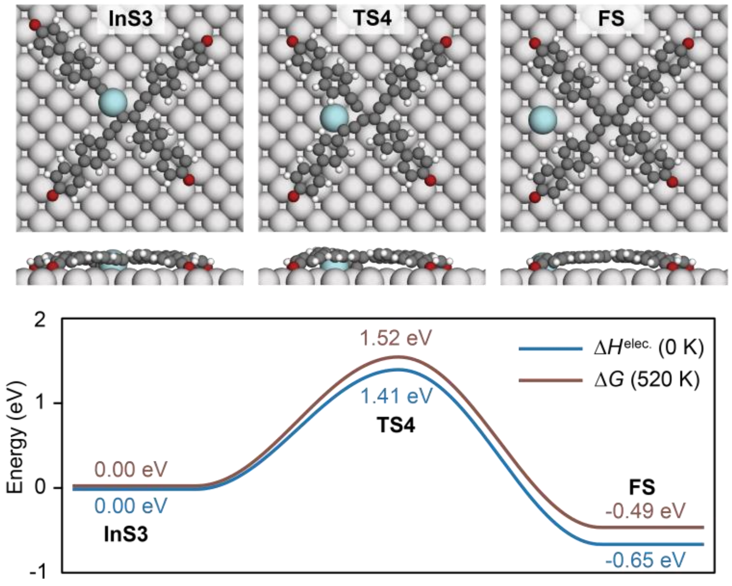

Supplementary Figure 16. Temperature-dependent calculation of the rate-limiting step in the reaction pathway. Energy profiles of the last Ag-removal step are shown, comparing the electronic enthalpy ($\Delta H^{\mathrm{elec.}}$) to the free energy ($\Delta G$) at 520 K. The free energy was calculated within the harmonic approximation, adding vibrational enthalpy and entropy to the electronic enthalpy. For the 0 K electronic enthalpy, the barrier from InS3 to FS is 1.41 eV, as shown in Figure 6 in the main text. Essentially, the barrier becomes slightly larger (1.52 eV) when the temperature reaches the 520 K set value, while remaining small enough allow for the identified coupling reaction. Source data are provided as a Source Data file.

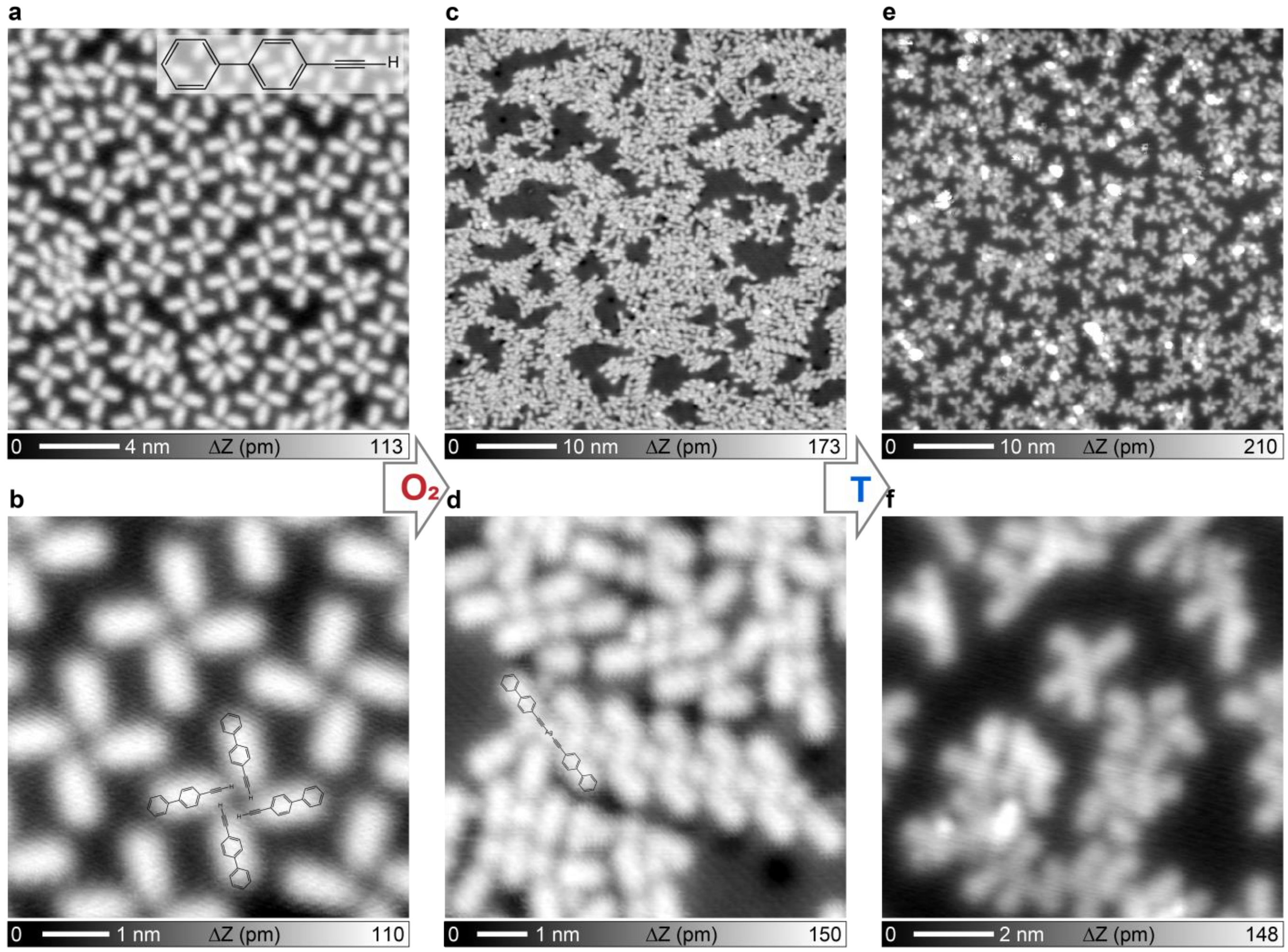

Supplementary Figure 17. Reaction scenario of EBP on Ag(100). (a-b) Large-scale and high-resolution STM images of the assembly structures after deposition of 4-Ethynylbiphenyl (EBP) molecules on Ag(100) substrate held at RT. Given the single alkynyl termination, the formed tetramer clusters aggregate without long-range order. (c-d) Without the interplay between hydroxyls, no ordered organometallic structures are found on the substrate after O_2 exposure. (e-f) Upon annealing the O_2-treated substrate at 500 K, the non-uniform addition products are scattered on the surface. Measurement parameters: (a) and (b) I_t = 1 nA, V_s = -100 mV; (c) I_t = 100 pA, V_s = -100 mV; (d) I_t = 500 pA, V_s = 10 mV; (e) and (f) I_t = 100 pA, V_s = 100 mV.

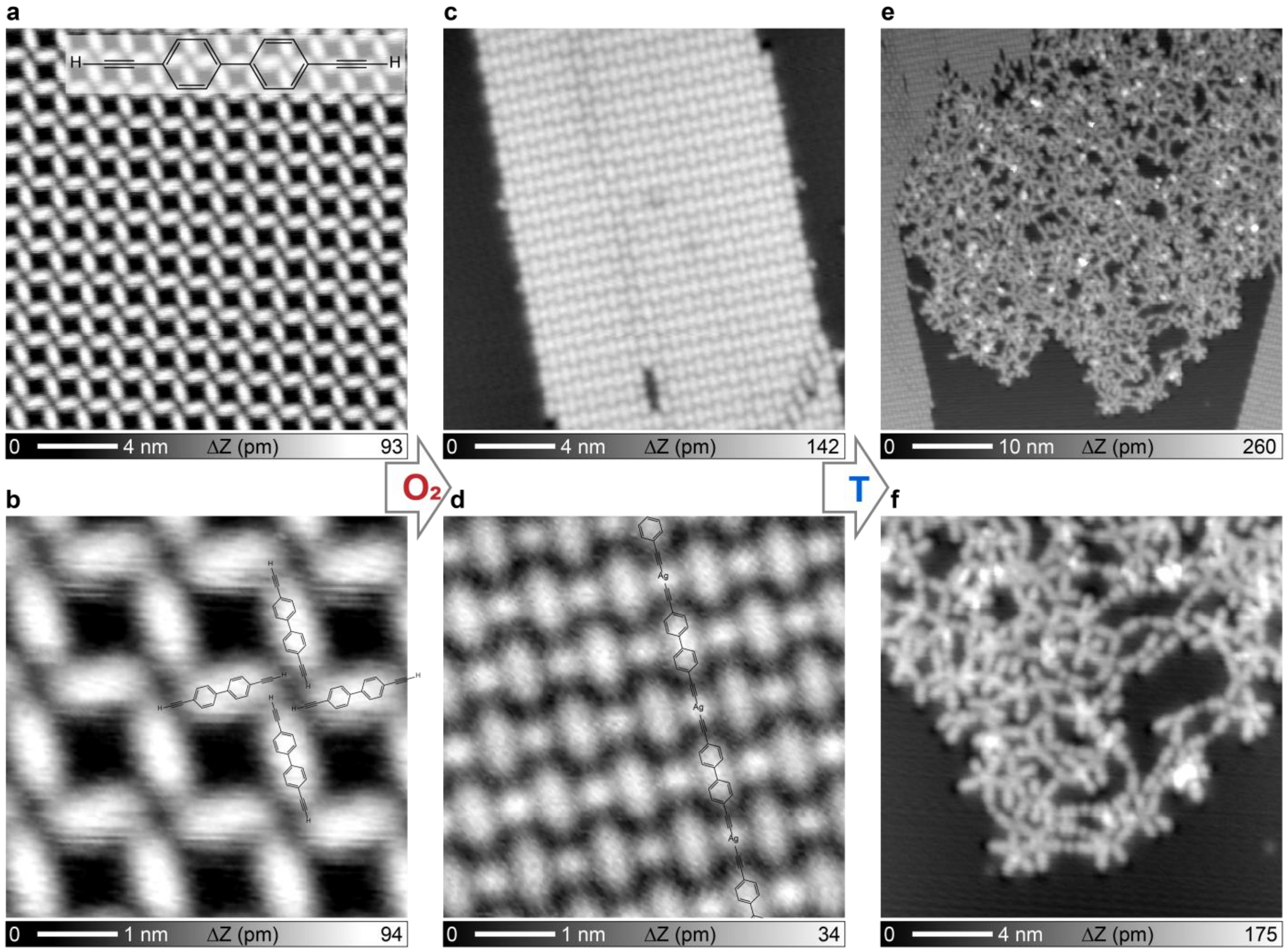

Supplementary Figure 18. Reaction scenario of DEBP on Ag(100). (a-b) Large-scale and high-resolution STM images of the rhombic network after deposition of 4,4'-diethynylbiphenyl (DEBP) molecules on Ag(100) substrate held at RT. (c-d) After exposing the rhombic network to O_2 at RT, a close-packed domain of organometallic chains evolves, reproducing the results reported recently[6]. (e-f) Upon annealing the O_2-treated substrate at 500 K, disordered structures emerge. Due to the reactivity of the alkynyl at both ends and the absence of hydroxyl directing groups, the products are very disordered and pure enetriyne connections are scarce. Measurement parameters: (a) I_t = 1 nA, V_s = 100 mV; (b-d) and (f) I_t = 1 nA, V_s = 10 mV; (e) I_t = 1 nA, V_s = -100 mV.

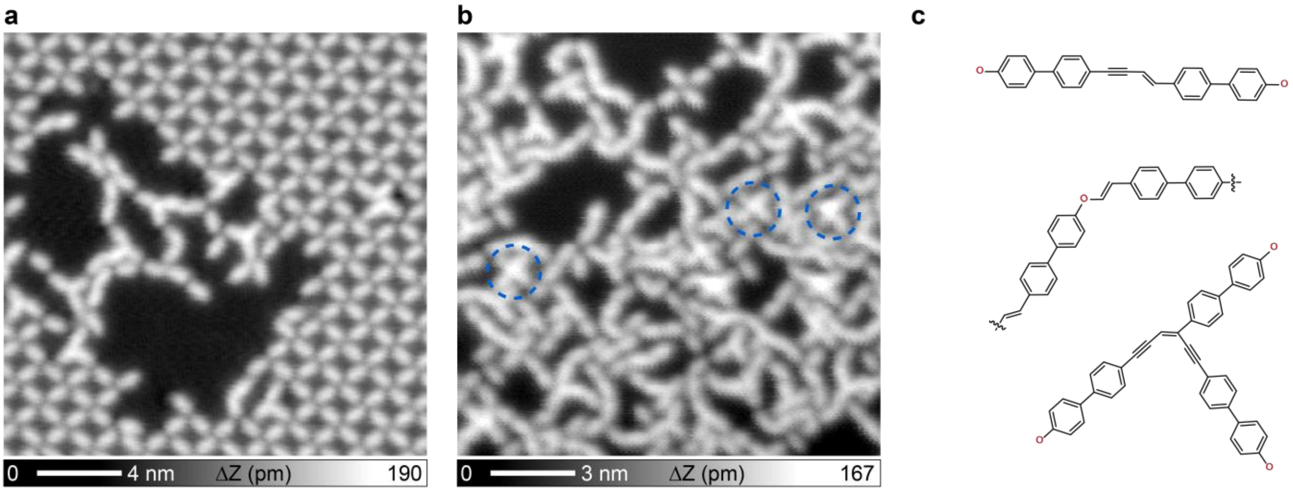

Supplementary Figure 19. Comparative experiment of annealing a EHBP layer on Ag(100) without previous O_2 exposure. Deposition of EHBP molecules with medium coverage on Ag(100) substrate held at RT gives rise to an ordered rhombic network, identical to the one in Figure 2c in the main text. (a) Annealing the substrate at 370 K induces formation of few short polymers and trimeric species at the edge of the rhombic network. (b) After annealing at 420 K, irregular structures and tetramer-like species (marked by blue dashed circles) can be found occasionally. (c) The hypothesized chemical structures of side products. This result indicates that O_2 exposure is crucial for the formation of enetriynes. Measurement parameters: (a) and (b) I_t = 1 nA, V_s = -100 mV.

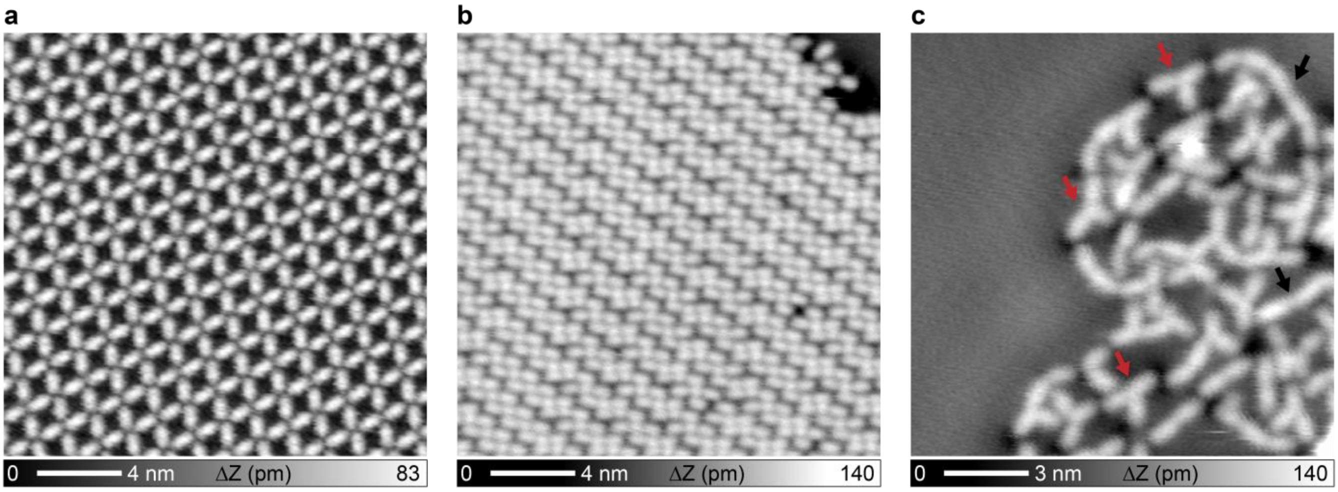

Supplementary Figure 20. Reaction scenario of EHBP on Ag(111). (a) STM image of the rhombic network after deposition of EHBP molecules with medium coverage on Ag(111) substrate held at RT. The unit cell of the network is similar to the values measured for the network on Ag(100). After exposing the rhombic network to O_2 (~450 L) at RT, a close-packed domain of organometallic dimers evolves, shown in (b). Note that the dimers are different from those in arrays obtained on Ag(100). Upon annealing the O_2-treated substrate at 470 K disordered structures evolve. A close-up image in (c) shows trimeric species (red arrows) and short polymers (black arrows) in the obtained irregular aggregates. This result indicates that the Ag(100) substrate symmetry plays an important role for directing the addition reaction affording

enetriynes. Measurement parameters: (a) I_t = 100 pA, V_s = -100 mV; (b) I_t = 1 nA, V_s = -100 mV; (c) I_t = 100 pA, V_s = -500 mV.

Supplementary references

1. Yang, B., *et al.* Intermediate states directed chiral transfer on a silver surface. *J. Am. Chem. Soc.* **141**, 168-174 (2019).
2. Li, Q., *et al.* Surface-controlled mono/diselective *ortho* C-H bond activation. *J. Am. Chem. Soc.* **138**, 2809-2814 (2016).
3. Giovanelli, L., *et al.* Combined photoemission spectroscopy and scanning tunneling microscopy study of the sequential dehydrogenation of hexahydroxytriphenylene on Ag(111). *J. Phys. Chem. C* **118**, 14899-14904 (2014).
4. Feng, L., *et al.* Supramolecular tessellations at surfaces by vertex design. *ACS Nano* **13**, 10603-10611 (2019).
5. Wang, T., *et al.* Unravelling the mechanism of Glaser coupling reaction on Ag(111) and Cu(111) surfaces: A case for halogen substituted terminal alkyne. *J. Phys. Chem. C* **122**, 14537-14545 (2018).
6. Zhang, C., *et al.* Steering the reaction pathways of terminal alkynes by introducing oxygen species: from C–C coupling to C–H activation. *J. Am. Chem. Soc.* **144**, 10282-10290 (2022).
